# Serum Vitamin A Is Associated with Variations in the Relationship between Plasma B6 Vitamers and Cardiovascular Disease Risk

**DOI:** 10.1016/j.tjnut.2025.10.036

**Published:** 2025-10-30

**Authors:** Indu Dhar, Gard FT Svingen, Arve Ulvik, Espen Ø Bjørnestad, Jørn V Sagen, Ottar K Nygård

**Affiliations:** 1 Mohn Nutrition Research Laboratory, Department of Clinical Science, University of Bergen, Bergen, Norway; 2 Centre for Nutrition, Department of Clinical Medicine, University of Bergen, Bergen, Norway; 3 Department of Heart Disease, Haukeland University Hospital, Bergen, Norway; 4 Bevital AS, Bergen, Norway; 5 Department of Cardiology, Stavanger University Hospital, Stavanger, Norway; 6 Division of Diagnostics and Technology, Akershus University Hospital, Lørenskog, Norway; 7 Faculty of Medicine, Institute of Clinical Medicine, University of Oslo, Oslo, Norway

**Keywords:** vitamin A, vitamin B6, acute myocardial infarction, lipids, atherosclerosis

## Abstract

**Background:**

Low concentrations of biologically active B6 vitamer, pyridoxal 5'-phosphate (PLP) are associated with an increased risk of cardiovascular disease (CVD). Vitamin A (Vit-A) promotes lipid homeostasis and the transport cholesterol. Vit-A may also stimulate the intracellular transport of PLP.

**Objectives:**

This study aimed to investigate whether Vit-A is associated with variations in the relationship of systemic B6-vitamers with incident acute myocardial infarctions (AMIs).

**Methods:**

A total of 4091 patients undergoing elective coronary angiography for suspected stable angina pectoris were studied. Associations of different plasma B6 vitamers, including PLP, pyridoxal (PL), 4-pyridoxic acid (PA), and PA/PL ratio with the risk of AMI according to median concentrations of Vit-A, were explored in Cox regression models.

**Results:**

Serum Vit-A demonstrated positive associations with PLP and PA/PL ratio at baseline (*P <* 0.001 for both). During a median follow-up of 7.5 y, 521 (12.7%) patients suffered an AMI. In age and sex-adjusted analyses, plasma PLP, PA, and PA/PL ratio showed an overall association with incident AMI {hazard ratio (HR) [95% confidence interval (CI)] per SD: 0.90 [0.82, 0.99; *P* = 0.02], 1.14 [1.05, 1.23; *P <* 0.001], and 1.28 [1.18, 1.39; *P <* 0.001], respectively}. However, low plasma PLP and high PA/PL ratio were associated with an increased risk of AMI primarily among patients with high compared with low Vit-A concentrations [HR (95% CI) per SD: 0.77 (0.68, 0.88; *P* < 0.001, *P*-interaction = 0.002) and 1.36 (1.23, 1.49; *P <* 0.001, *P*-interaction = 0.05), respectively]. The interactions persisted after multivariable adjustment (both *P-*interactions ≤ 0.04).

**Conclusions:**

The relationship between vitamin B6 indexes and AMI risk varied according to serum Vit-A concentrations. Additional research is needed to clarify the importance of Vit-A and B6 bioavailability in atherosclerotic CVD.

This trial was registered at clinicaltrials.gov as NCT00354081.

## Introduction

Vitamin B6 (Vit-B6) is a water-soluble nutrient that serves as a cofactor for >100 human enzymatic reactions involved in the metabolism of amino acids, carbohydrates, neurotransmitters, and lipids [1]. The major forms of B6 vitamers found in plasma include the active form, pyridoxal 5′-phosphate (PLP); the transport form, pyridoxal (PL); and the catabolic inactive end product, 4-pyridoxic acid (PA) [1]. Observational studies have shown that low circulating PLP is associated with higher risk of cardiovascular disease (CVD) [2] and stroke [3], whereas higher plasma PA is positively correlated with CVD [4]. However, Vit-B6 treatment had no significant effect on major cardiovascular events in several randomized clinical trials aimed for secondary CVD prevention [5,6]. In contrast, one study found an increased risk of restenosis after percutaneous coronary intervention following administration of Vit-B6 together with folic acid and vitamin B12 [7].

Vitamin A (Vit-A as retinol) is a fat-soluble micronutrient obtained from the diet and is essential for diverse physiological functions. Vit-A is predominantly stored in the liver as retinyl esters, which can be mobilized and converted to retinol and then to retinal and subsequently to active form, retinoic acid (RA), which binds to nuclear RA receptors for signaling [8]. The role of Vit-A in the pathogenesis of CVD remains unclear [8]; however, available evidence suggests that Vit-A metabolism is related to lipid homeostasis and cholesterol transport. More specifically, *all-trans* RA induces hepatic glycine-N-methyl-transferase (GNMT) [9,10], and reduced GNMT may impair cholesterol export and induce hyperlipidemia [11]. Nevertheless, apart from this function, GNMT regulation may have amplified effects by influencing gene expression through epigenetic modifications [9]. Furthermore, *all-trans* RA stimulates monocyte differentiation [12] and enhances cholesterol efflux from macrophages via upregulating ATP-binding cassette transporters A1 (ABCA1) and G1 (ABCG1) expression [13,14], thus potentially suppressing macrophage foam cell formation and atherosclerosis development.

Notably, in addition to its role in regulating lipid metabolism, *all-trans* RA is also shown to upregulate both expression and activity of alkaline phosphatase (ALP) [15,16], which is necessary for the uptake of PLP from the circulation [1]. Studies also showed that aldehyde oxidase (AOX), a cytosolic enzyme believed to catalyze the irreversible oxidation of PL to PA [1], contributes to *all-trans* RA biosynthesis in humans [17]; thereby further linking Vit-A to intracellular PLP transport.

Taken together, these observations suggest a potential interaction between these 2 vitamins, which may be associated with lipid metabolism in the liver, circulation, endothelium, and monocytes, which in turn may relate to atherosclerotic CVD risk. However, to our knowledge, no data exist about this interaction in relation to CVD outcomes in either experimental or human settings. We assessed whether the associations of different Vit-B6 measures, including PLP, PL, PA, and PA/PL ratio with the risk of acute myocardial infarction (AMI), were influenced by serum concentrations of Vit-A in a large prospective cohort of patients with suspected stable angina pectoris (SAP) with long-term follow-up.

## Methods

### Study cohort

**FIGURE 1:**
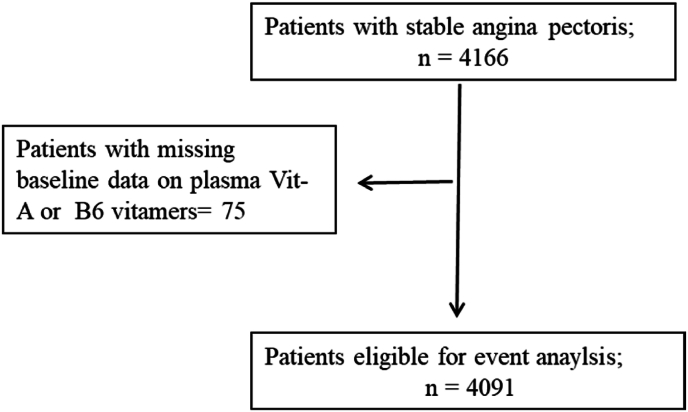
Flowchart showing patient selection.

The study population has been detailed elsewhere [18]. In short, 4166 adults, mainly white patients undergoing coronary angiography between January 2000 and April 2004 at 2 university hospitals in western Norway were studied. Among these, 2573 (61.8%) of patients were randomized in the Western Norway B Vitamin Intervention Trial (WENBIT) (clinicaltrials.gov Identifier: NCT00354081) [6]. Patients with missing data on circulating Vit-A or B6 vitamers were excluded, leaving 4091 subjects eligible for the current analyses (Figure 1). The study fulfilled the Declaration of Helsinki and was approved by the Regional Committee for Medical and Health Research Ethics, and the Norwegian Data Inspectorate. All study participants provided written informed consent.

### Baseline variables

Each study subject provided information about lifestyle, and medical history through self-administered questionnaires/interviews, and the information was verified against hospital records. Smoking status, hypertension, left ventricular ejection fraction, and angiographic extent of coronary artery disease were determined as previously described [19].

### Biochemical analyses

Blood samples were collected by study personnel before angiography and stored at −80°C until analysis in 2007. Serum Vit-A (as *all-trans-retinol*) and plasma concentrations of PLP, PL, and PA were analyzed by liquid chromatography/tandem mass spectrometry at BEVITAL AS, Bergen, Norway (www.bevital.no). Serum apolipoprotein (apo) A1, apoB100, and C-reactive protein (CRP) were analyzed and determined as previously described [19]. Serum LDL cholesterol was obtained using the Friedewald formula and the estimated glomerular filtration rate (eGFR) [20] was calculated using the Chronic Kidney Disease Epidemiology Collaboration formula.

### End points and follow-up

Study patients were followed from enrollment until they experienced an AMI (fatal or nonfatal) or throughout 2009. AMI was classified according to the International Classification of Disease, 10th Revision System. We obtained information on clinical events from the cardiovascular disease in Norway project (CVDNOR; https://cvdnor.b.uib.no/) as previously described [21].

### Statistical methods

Continuous variables are presented as medians (5th–95th percentiles) and categorical variables are reported as counts (%). The association of serum Vit-A with several baseline continuous and categorical covariates was assessed with unadjusted linear or logistic regression. Cox regression models were used to calculate hazard ratios (HRs) and 95% confidence intervals (CIs) for incident AMI as per 1-SD increment in log-transformed plasma PLP, PA, PL, and PL/PA ratio. Proportionality assumptions were tested by calculating Schoenfeld and scaled Schoenfeld residuals. A simple model (model 1) was adjusted for age (continuous) and sex. Covariates in the multivariable-adjusted model (model 2) additionally included BMI (continuous), diabetes mellitus (yes/no), current smoking (yes/no), previous myocardial infarction (yes/no), apoA1, and apoB100 (both continuous). Because altered Vit-B6 metabolism has been associated with inflammation [1] and renal insufficiency [22], we additionally included CRP and eGFR in model 2. Adjustments for hypertension, or B-vitamin supplementation at baseline did not materially influence the risk associations and were excluded in the final model (data not shown).

Serum Vit-A was grouped according to median values, and the possible interactions with B-6 vitamers on incident AMI risk were tested by including interaction product terms to the Cox models.

All probability values were 2-sided and considered significant when <0.05. Statistical analyses were performed using SPSS 28 (SPSS Inc).

## Results

### Baseline characteristics

The median (5th–95th percentile) age for the 4091 patients at baseline was 62 (44–78) y, and approximately 72.0% were male (Table 1). As presented in Table 1, patients who were male had higher serum Vit-A concentrations. Furthermore, we observed a positive association of Vit-A with BMI, neopterin, and most serum lipid parameters, whereas there was an inverse association with CRP and eGFR. Serum Vit-A was positively associated with PLP, the PA/PL ratio, and 3-methyl-histidine (3-mHT) (Table 1), irrespective of adjustments for protein intake (data not shown).

**TABLE 1:** Baseline characteristics of the patients in the total population and according to serum vitamin A median levels (2.82 μmol/L)

	Total (*n* = 4091)	Vit-A median levels (μmol/L)		*P* value
		≤Median (2.82) (*n* = 2044)	>Median (2.82) (*n* = 2047)	
Serum Vit-A (μmol/L)	2.82 (1.96–4.15)	2.45 (1.81–2.78)	3.29 (2.86–4.56)	–
Age (y)	62 (44–78)	63 (44–78)	61(44–78)	<0.001
Male sex, n (%)	2939 (71.8)	1438 (70.4)	1501 (73.3)	0.034
BMI (kg/m^2^)	26 (21–33)	26 (20–33.7)	26 (21–33)	0.01
Coronary risk factors, *n* (%)				
Hypertension	1916 (46.8)	929 (45.5)	987 (48.2)	0.076
Diabetes mellitus	484 (11.8)	252 (12.3)	232 (11.3)	0.32
Current smoking	1298 (31.7)	670 (32.8)	628 (30.7)	0.15
Prior AMI	1645 (40.2)	826 (40.4)	819 (40.0)	0.79
eGFR (mL/min per 1.73 m^2^)	91 (57–111)	92 (63–112)	88 (49–109)	<0.001
Serum CRP (mg/L)	1.78 (0.35–12.6)	1.95 (0.35–16.5)	1.64 (0.35–9.59)	<0.001
Serum neopterin (nmol/L)	8.18 (5.15–16.3)	7.96 (5.07–15.3)	8.42 (5.27–17.5)	<0.001
LVEF (%)	65 (40–80)	65 (40–80)	66 (40–80)	0.99
3-mHT (μmol/L)	2.78 (0.73–14.7)	2.59 (0.68–13.6)	2.96 (0.82–15.8)	<0.001
Extent of CAD, *n* (%)				<0.001
No stenotic vessels	1033 (25.3)	445 (21.8)	588 (28.7)	
1-vessel disease	940 (23.0)	449 (22.0)	491 (24.0)	
2-vessel disease	916 (22.4)	489 (23.9)	427 (20.9)	
3-vessel disease	1202 (29.4)	661 (32.3)	541 (26.4)	
Plasma B6 vitamers				
PLP (nmol/L)	41.3 (18.6–124)	37.5 (17.5–107)	45.5 (19.6–140)	<0.001
PL (nmol/L)	9.53 (5.15–31.8)	8.97 (4.95–26.6)	10.2 (5.46–36.0)	0.93
PA (nmol/L)	24.4 (13.6–95.0)	22.9 (12.9–76.5)	26.8 (14.5–115)	0.09
PA/PL ratio	2.61 (1.52–4.69)	2.56 (1.49–4.52)	2.66 (1.54–4.94)	<0.001
Serum lipid parameters				
Total cholesterol (mmol/L)	4.90 (3.5–7.1)	4.80 (3.40–6.98)	5.10 (3.60–7.30)	<0.001
LDL-C (mmol/L)	2.90 (1.70–5.0)	2.90 (1.70–4.80)	3.0 (1.76–5.04)	0.001
HDL-C (mmol/L)	1.20 (0.80–2.0)	1.20 (0.80–2.0)	1.20 (0.80–2.0)	0.02
Triglycerides (mmol/L)	1.50 (0.70–3.64)	1.33 (0.66–3.19)	1.68 (0.75–4.06)	<0.001
ApoB100 (g/L)	0.87 (0. 57–1.36)	0.85 (0.56–1.31)	0.89 (0.58–1.39)	<0.001
Apo A1 (g/L)	1.30 (0.92–1.80)	1.26 (0.89–1.74)	1.33 (0.96–1.85)	<0.001
WENBIT B-vitamin treatment^1^ (daily dose), *n* (%)				0.28
FA (0.8 mg) +B12 (0.4 mg) +B6 (40 mg)	633 (15.5)	341 (16.7)	292 (14.3)	
FA (0.8 mg) +B12 (0.4 mg)	629 (15.4)	360 (17.6)	269 (13.1)	
B6(40 mg)	630 (15.4)	359 (17.6)	271 (13.2)	
Placebo	630 (15.4)	360 (17.6)	270 (13.2)	
Medications after angiography, *n* (%)				
Aspirin	3331 (81.4)	1683 (82.3)	1648 (80.5)	0.13
Statins	3272 (80.0)	1648 (80.6)	1624 (79.3)	0.30
β-blocker	2963 (72.4)	1497 (73.2)	1466 (71.6)	0.25

Continuous variables are presented as medians (5th–95th percentile) and categorical variables are reported as counts (%).

Abbreviations: 3-mHT, 3-methylhistidine; AMI, acute myocardial infarction; apoA1, apolipoprotein A1; apoB100, apolipoprotein B100; B6, vitamin B6; B12, vitamin B12; BMI, body mass index; CAD, coronary artery disease; CRP, C-reactive protein; eGFR, estimated glomerular filtration rate; FA, folic acid; HDL-C, high-density lipoprotein cholesterol; LDL-C, low-density lipoprotein cholesterol; LVEF, left ventricular ejection fraction; PA, pyridoxic acid; PL, pyridoxal; PLP, pyridoxal 5′ phosphate; Vit-A, vitamin A; WENBIT, Western Norway B Vitamin Intervention Trial.

1 Treatment: January 2000-October 2005.

### Associations of B6 vitamers with the risk of AMI according to serum Vit-A concentrations

A total of 521 (12.7%) patients experienced an AMI during a median follow-up time of 7.5 y. Plasma PLP showed an inverse association with AMI in the age and sex-adjusted model, but not after adjusting for traditional CVD risk factors (Table 2). There were also positive relationships between the PA and PA/PL ratio with AMI, which were slightly attenuated after multivariable adjustments (Table 2).

**TABLE 2:** Risk association between plasma vitamin B6 indexes (PLP, PA, PL, and PL/PA ratio) and incident acute myocardial infarction

B6 indexes	Model 1 HR (95% CI )^1^	*P* value	Model 2 HR (95% CI )^1^	*P* value
PLP	0.90 (0.82, 0.99)	0.02	0.97 (0.89, 1.06)	0.55
PL	1.01 (0.93, 1.10)	0.84	1.03 (0.95, 1.12)	0.42
PA	1.14 (1.05, 1.23)	<0.001	1.11 (1.03, 1.20)	0.01
PA/PL ratio	1.28 (1.18, 1.39)	<0.001	1.18 (1.07, 1.29)	<0.001

Model 1 was adjusted for age and sex. Model 2 was additionally adjusted for body mass index, diabetes mellitus, current smoking, previous acute myocardial infarction, apolipoprotein A1, apolipoprotein B100, C-reactive protein and estimated glomerular filtration rate.

Abbreviations: CI, confidence interval; HR, hazard ratio; PA, pyridoxic acid; PL, pyridoxal; PLP, pyridoxal 5′-phosphate.

1 Per 1-SD increase in log-transformed concentrations.

The age and sex-adjusted risk associations between several Vit-B6 indexes and incident AMI according to Vit-A concentrations are presented in Table 3. We observed a stronger inverse association between PLP and AMI among patients with high (>median) Vit-A concentrations [HR (95% CI) per SD: 0.77 (0.68, 0.88)], whereas there was no relationship among those with low (≤median) Vit-A concentrations (*P*-interaction = 0.002) (Table 3). Additionally, we found a stronger positive relationship of plasma PA/PL ratio with AMI in patients with high [HR (95% CI) per SD: 1.36 (1.23, 1.49)] compared with low serum Vit-A concentrations (*P*-interaction = 0.05) (Table 3). These interactions remained significant after multivariable adjustment (both *P*-interaction ≤ 0.04) (Table 4), and excluding patients treated with Vit-B6 in WENBIT or those with kidney dysfunction (data not shown).

**Table 3:** Age and sex-adjusted risk association between plasma vitamin B6 indexes and acute myocardial infarction according to median serum vitamin A levels (2.82 μmol/L)

	Serum vitamin A				
	≤Median		>Median		
*N*	2044		2047		
Events	267		254		
B6 indexes	HR (95% CI) 1	*P*-value	HR (95% CI) 1	*P-*value	*P-int*
PLP	1.03 (0.91-1.16)	0.69	0.77 (0.68-0.88)	<0.001	0.002
PL	1.09 (0.98-1.22)	0.09	0.90 (0.78-1.04)	0.16	0.04
PA	1.17 (1.06-1.30)	0.003	1.10 (0.99-1.23)	0.09	0.48
PA/PL ratio	1.17 (1.03-1.33)	0.02	1.36 (1.23-1.49)	<0.001	0.05

HR (95% CI) were estimated by Cox regression model.

Abbreviations: PA, pyridoxic acid; PL pyridoxal, PLP, pyridoxal 5´-phosphate.

1 Per 1-SD increase in log-transformed concentrations

**Table 4:** Multivariable-adjusted risk association between plasma vitamin B6 indexes and acute myocardial infarction according to median serum vitamin A levels (2.82 μmol/L).

	Vitamin A				
	≤ median		>median		
B6 indexes	HR (95% CI) 1	*P* value	HR (95% CI) 1	*P* value	*P-int*
PLP	1.10 (0.97-1.23)	0.13	0.87 (0.76-0.99)	0.04	0.01
PL	1.11 (1.01-1.23)	0.03	0.94 (0.81-1.07)	0.34	0.10
PA	1.16 (1.05-1.29)	0.003	1.03 (0.91-1.17)	0.61	0.61
PA/PL ratio	1.10 (0.96-1.26)	0.16	1.20 (1.06-1.36)	0.005	0.04

HR (95% CI) was estimated by the Cox regression model, adjusted for age, sex, body mass index, diabetes mellitus, current smoking, previous acute myocardial infarction, apolipoprotein A1, apolipoprotein B100, C-reactive protein, and estimated glomerular filtration rate.

Abbreviations: CI, confidence interval; HR, hazard ratio; PA, pyridoxic acid; PL, pyridoxal; PLP, pyridoxal 5′-phosphate.

1 Per 1-SD increase in log-transformed concentrations.

## Discussion

### Principal findings

In this prospective study among 4091 patients undergoing coronary angiography for suspected SAP, low plasma PLP, and a high PA/PL ratio were associated with an increased risk of incident AMI primarily in patients with elevated Vit-A concentrations.

### Vit-B6 indexes, Vit-A, and CVD

Numerous epidemiologic studies have related low Vit-B6 intake to an elevated CVD risk [23,24]. These observations were further corroborated by reports of reduced circulating PLP concentrations in patients subsequently experiencing cardiovascular conditions [25]. In line, several prior studies also reported a negative association between plasma PLP and CVD risk [2,3]. Our current study is concordant with these findings but also extends them by demonstrating that such an association may be present primarily in patients with elevated Vit-A. We also showed that the association of PA/PL ratio, a parameter of Vit-B6 catabolism [26], with the risk of AMI was particularly stronger in patients with elevated Vit-A.

### Possible mechanisms

In our study, Vit-A concentrations were positively associated with PLP and PA/PL ratio as well as 3-mHT, a marker of high meat (protein) intake [27], which, in turn, is positively related to Vit-B6 status [26]. However, the significant positive associations of serum Vit-A with PLP and PA/PL ratio and 3-mHT persisted after adjusting for protein intake, indicating that high levels of these parameters do not solely reflect increased protein consumption. Alternatively, 3-mHT serves as a valuable marker of increased muscle protein breakdown [27], which is associated with increased PLP turnover [28]. This could suggest a role of Vit-A in the intracellular transport of PLP during muscle proteolysis. Indeed, available evidence suggests that *all-trans* RA stimulates ALP [15,16], an isoenzyme that hydrolyzes PLP. Others have shown that the AOX gene involved in the irreversible oxidation of PL to PA [1], also contributes to *all-trans* RA synthesis [17]. These findings may explain the stronger association of low plasma PLP and/or high PA/PL ratio with increased AMI risk among patients with elevated Vit-A concentrations.

Lipid peroxidation is regarded as a fundamental and causal mechanism in atherosclerosis development [29]. PLP downregulation is well known in inflammation [1]; however, treatment by Vit-B6 failed to reduce CVD risk in secondary prevention trials [5], including the WENBIT [6], which constituted the main proportion of this study cohort. Notably, PLP to PL oxidation in plasma releases inorganic phosphate (Pi) [1], which may potentially be transported into cells and used for ATP formation [30,31] and thereby consequent phosphorylation reactions [31,32] rather than being reconverted to PLP intracellularly. For instance, by NAD kinases to produce NADP (H), which is a vital cofactor in nucleotide and lipid synthesis, including cholesterol [32]. This may imply an increased intracellular demand for Pi, and consequent PL catabolism in need of Pi for the various NADP-related mechanisms. In addition, the glycine cleavage system, the main pathway regulating glycine catabolism, is a PLP-dependent enzyme [1], suggesting that increased PLP catabolism may conserve glycine. Excess glycine may be exported to the cytosol and used for GNMT reactions, implicated in cholesterol homeostasis [11,33]. We have also previously shown that high plasma glycine is inversely associated with AMI risk [34]. Thus, inhibiting glycine cleavage system via increased PLP catabolism may theoretically be linked to an upregulation of substrates for the GNMT flux. Importantly, diminished endothelial GNMT has been associated with oxidized-LDL accumulation [33], which is strongly enriched in oxysterols [35], which in turn stimulate the production of *all-trans* RA [36]. However, the relationship between Vit-A and GNMT is complex, and *all-trans* RA induces hepatic GNMT [9,10], thereby creating a protective feedback mechanism. These observations indicate that Vit-A synthesis may be a feedback response to decreased lipid flux between the liver and peripheral tissues and that Vit-A production may partly be induced to promote intracellular catabolism of PLP and PL, which could serve as counterregulatory protective mechanisms to help restore cholesterol levels in tissues.

In addition, *all-trans* RA is reported to increase ABCA1/G1 expression and cholesterol efflux to apo A1 and HDL in macrophages [13,14]. In a subsample of the same study population, we previously showed that low apoA1, the major protein of HDL, was associated with increased AMI risk primarily in those with high Vit-A [37]. This may suggest that reduced export of cholesterol from monocytes to HDL particles due to apoA1 deficiency contributes to atherosclerosis in these patients. Furthermore, activated monocytes/macrophages upon stimulation by interferon-γ secrete neopterin, which is also shown to enhance macrophage cholesterol efflux and reduce atherosclerosis [38]. We demonstrated recently that high neopterin concentrations were predictive of AMI risk in patients with elevated Vit-A [39], further indicating that increased Vit-A together with neopterin may represent a feedback response aiming to restore cholesterol transport and homeostasis in tissues.

### Strengths and limitations

The major study strengths are its long-term follow-up, large study sample, and detailed information on patient baseline characteristics.

However, certain limitations are addressed, including the observational design of our study that cannot be used to infer causal direction. Second, we did not have information on the active form of Vit-A, or on retinol-binding proteins such as RBP-4, which have been associated with CVD risk [8]; therefore, we could not estimate retinoid metabolism and future research are required to address this gap. Third, the systemic metabolite concentrations were measured only at baseline, preventing the assessment of possible fluctuations during the course of follow-up. However, both circulating Vit-A and B6 vitamers have fair-to-good within-subject reproducibility, allowing 1-timepoint measurement of biomarker status [40]. Fourth, Vit-A [41] and PA [22] concentrations are reported to be elevated in patients with renal dysfunction. The majority of our study population had normal renal function (with a median eGFR of 91 mL/min per 1.73 m²), which could thus limit the generalizability of our findings to patients with renal disease. However, the interaction terms between Vit-A and PA/PL ratio remained significant even after adjustment for eGFR or excluding patients with severe kidney dysfunction. This suggests that the current findings are not mediated solely by renal impairment and thereby argue against non-generalizability. Finally, the mechanisms postulated in this study are purely speculative and as the data in this study are observational, further studies are needed to evaluate whether these associations reflect underlying biological processes.

### Conclusions

In patients with suspected SAP, low PLP concentrations and an increased PA/PL ratio were associated with long-term risk of AMI, primarily in patients with elevated Vit-A concentrations. Further studies are also required to gain greater insight into the pathological importance of Vit-A and B6 bioavailability in atherothrombosis.

## Author contributions

The authors’ responsibilities were as follows – ID, OKN: contributed to the conception and data acquisition; ID: designed the work, performed analysis, interpreted the data, and drafted the manuscript; ID, GFTS, AU, EØB, JVS, OKN: contributed to the data interpretation; ID, GFTS, AU, EØB, JVS, OKN: contributed to the critical revision of the manuscript for intellectual content; and all authors: gave final approval and agreed to be accountable for all aspects of the work ensuring integrity and accuracy.

## Data availability

Data described in the manuscript, code book, and analytic code will be made available upon reasonable request from the corresponding author.

## Funding

This work was funded by the University of Bergen, the Department of Heart Disease at Haukeland University Hospital, the Western Norway Regional Health Authority, the Trond Mohn Foundation (BFS2017NUTRITIONLAB), and the Foundation to Promote Research into Functional Vitamin B12 Deficiency, Bergen, Norway.

## Conflict of interest

The authors report no conflicts of interest.
